# Clinicopathological features and cancer-specific survival in young patients with ulcerative colitis–associated colorectal neoplasia: a nationwide cohort study in Japan

**DOI:** 10.1093/ibd/izag069

**Published:** 2026-05-19

**Authors:** Takahide Shinagawa, Koichi Komatsu, Motoi Uchino, Hiroki Ikeuchi, Kohei Shigeta, Shiro Oka, Daijiro Higashi, Shimpei Ogawa, Kazuhiro Watanabe, Masatsune Shibutani, Yoshiki Okita, Toshifumi Wakai, Yusuke Mizuuchi, Kinya Okamoto, Kazutaka Yamada, Yu Sato, Takayuki Ogino, Hideaki Kimura, Kenichi Takahashi, Koya Hida, Yusuke Kinugasa, Fumio Ishida, Junji Okuda, Koji Daito, Takayuki Yamamoto, Seiichiro Yamamoto, Fumikazu Koyama, Junichiro Hiro, Koji Komori, Dai Shida, Junya Arakaki, Fumihiko Fujita, Takatoshi Matsuyama, Hideki Ueno, Hiroki Ochiai, Atsuo Maemoto, Riichiro Nezu, Shin Sasaki, Eiji Sunami, Tatsuki Noguchi, Kenichi Sugihara, Yoichi Ajioka, Soichiro Ishihara, the Study Group for Inflammatory Bowel Disease Associated Intestinal Cancers by Japanese Society for Cancer of the Colon and Rectum, Soichiro Ishihara, Tanaka Shinji, Uraoka Toshio, Saito Yutaka, Takamaru Hiroyuki, Nakase Hiroshi, Naganuma Makoto, Hisamatsu Tadakazu, Fujii Toshimitsu, Matsuura Minoru, Matsumoto Takayuki, Kenji Watanabe, Hiroki Ikeuchi, Motoi Uchino, Yoshiki Okita, Okabayashi Koji, Kazutaka Koganei, Sugita Akira, Kenichi Takahashi, Hata Keisuke, Daijiro Higashi, Futami Kitaro, Matsuda Keiji, Mizushima Tsunekazu, Watadani Yusuke, Kawachi Hiroshi, Shimoda Masayuki, Sugai Tamotsu, Yoshida Masahiro, Yamaguchi Naohiko, Itabashi Michio, Anzai Hiroyuki, Kishikawa Junko, Koichi Komatsu, Takahide Shinagawa, Tsushima Tatsuya, Tatsuki Noguchi, Okada Satoshi, Kenichi Sugihara, Yoichi Ajioka

**Affiliations:** Department of Surgical Oncology, The University of Tokyo, Tokyo, Japan; Department of Surgical Oncology, The University of Tokyo, Tokyo, Japan; Department of Inflammatory Bowel Disease Surgery, Hyogo Medical University, Nishinomiya, Japan; Department of Inflammatory Bowel Disease Surgery, Hyogo Medical University, Nishinomiya, Japan; Department of Surgery, Keio University School of Medicine, Tokyo, Japan; Department of Gastroenterology, Graduate School of Biomedical and Health Sciences, Hiroshima University, Hiroshima, Japan; Department of Surgery, Fukuoka University Chikushi Hospital, Chikushino, Japan; Department of Surgery, Institute of Gastroenterology, Tokyo Women’s Medical University, Tokyo, Japan; Department of Surgery, Tohoku University Graduate School of Medicine, Sendai, Japan; Department of Gastroenterological Surgery, Osaka Metropolitan University Graduate School of Medicine, Osaka, Japan; Department of Gastrointestinal and Pediatric Surgery, Institute of Life Sciences, Mie University Graduate School of Medicine, Tsu, Japan; Division of Digestive and General Surgery, Graduate School of Medical and Dental Sciences, Niigata University, Niigata, Japan; Department of Surgery and Oncology, Graduate School of Medical Sciences, Kyushu University, Fukuoka, Japan; Department of Coloproctology, Tokyo Yamate Medical Center, Tokyo, Japan; Department of Surgery, Coloproctology Center Takano Hospital, Kumamoto, Japan; Department of Surgery, Toho University Sakura Medical Center, Chiba, Japan; Department of Gastroenterological Surgery, Graduate School of Medicine, Osaka University, Osaka, Japan; Inflammatory Bowel Disease Center, Yokohama City University Medical Center, Yokohama, Japan; Department of Colorectal Surgery, Tohoku Rosai Hospital, Sendai, Japan; Department of Surgery, Kyoto University Hospital, Kyoto, Japan; Department of Gastrointestinal Surgery, Institute of Science Tokyo, Tokyo, Japan; Digestive disease center, Showa University Northern Yokohama Hospital, Kanagawa, Japan; Department of General and Gastroenterological Surgery, Osaka Medical and Pharmaceutical University, Takatsuki, Japan; Department of Surgery, Faculty of Medicine, Kindai University, Osaka, Japan; Inflammatory Bowel Disease Center, Yokkaichi Hazu Medical Center, Yokkaichi, Japan; Department of Gastroenterological Surgery, Tokai University School of Medicine, Tokyo, Japan; Department of Surgery, Nara Medical University, Nara, Japan; Department of Surgery, Fujita Health University, School of Medicine, Aichi, Japan; Department of Gastroenterological Surgery, Aichi Cancer Center Hospital, Aichi, Japan; Department of Surgery, IMSUT Hospital, The Institute of Medical Science, The University of Tokyo, Tokyo, Japan; Center for Gastroenterology, Department of Surgery, Urasoe General Hospital, Okinawa, Japan; Department of Surgery, Kurume University Hospital, Kurume, Japan; Department of Digestive Tract and General Surgery, Saitama Medical Center, Saitama, Japan; Department of Surgery, National Defense Medical College, Tokorozawa, Japan; Department of Surgery, Teikyo University School of Medicine, Tokyo, Japan; Inflammatory Bowel Disease Center, Sapporo Higashi Tokushukai Hospital, Sapporo, Japan; Department of Surgery, Nishinomiya Municipal Central Hospital, Nishinomiya, Japan; Department of Coloproctological Surgery, Japanese Red Cross Medical Center, Tokyo, Japan; Department of Surgery, Kyorin University, Tokyo, Japan; Department of Surgical Oncology, The University of Tokyo, Tokyo, Japan; Institute of Science Tokyo, Tokyo, Japan; Department of Pathology, Niigata University, Niigata, Japan; Department of Surgical Oncology, The University of Tokyo, Tokyo, Japan

**Keywords:** inflammatory bowel disease, ulcerative colitis, colitis-associated colorectal neoplasia, colorectal cancer, young-onset

## Abstract

**Background:**

The incidence of ulcerative colitis–associated colorectal neoplasia (UCAN) is increasing, but clinicopathological features and outcomes in young patients remain unclear. We aimed to clarify the characteristics and survival of UCAN diagnosed before the age of 40 using years in a nationwide Japanese cohort study conducted by the Japanese Society for Cancer of the Colon and Rectum.

**Methods:**

We retrospectively analyzed 1212 patients with UCAN treated at 43 institutions in Japan during the period from 1983 to 2020. Patients were categorized as young (<40 years) or non-young (≥40 years). Clinicopathological features, cancer-specific survival (CSS), relapse-free survival (RFS), and survival after recurrence (SAR) were compared.

**Results:**

Young-onset UCAN accounted for 252 patients (20.8%) and showed a shorter interval from UC onset to carcinogenesis (14.0 vs 17.0 years; *P < .*001), more frequent pancolitis (81.3% vs 74.8%; *P = .*012), and a higher proportion of poorly differentiated, mucinous, or signet-ring cell carcinoma (20.2% vs 13.3%; *P < .*001). Five-year CSS was significantly lower in young patients (85.1% vs 89.8%; *P = .*039), whereas RFS was comparable (86.2% vs 86.3%; *P = .*750). Minimally invasive surgery (MIS) was independently associated with improved CSS (hazard ratio [HR], 0.593; 95% CI, 0.356-0.987; *P = .*045). The SAR remained poor in both age groups (23.6% vs 28.5%; *P = .*468).

**Conclusions:**

When diagnosed in patients aged <40 years, UCAN is characterized by earlier carcinogenesis, extensive disease, and aggressive histology, with worse CSS. The potential role of MIS in improving prognosis warrants further prospective investigation, while the outcome of SAR remains poor irrespective of patient age.

Key Messages *What is already known?*

Patients with long-standing ulcerative colitis (UC) are at increased risk of developing UC–associated colorectal neoplasia (UCAN), but clinicopathological features and outcomes of UCAN in younger patients remain poorly defined.

*What is new here?*

In this nationwide cohort, UCAN in patients aged <40 years accounted for approximately 20% of cases and was characterized by earlier carcinogenesis, more extensive colitis, aggressive histology, and worse cancer-specific survival, while survival after recurrence remained poor. Minimally invasive surgery was associated with improved cancer-specific survival, although this may reflect differences in patient selection and disease stage.

*How can this study help patient care?*

These findings support tailored surveillance strategies and surgical management for patients with young-onset UCAN.

## Introduction

The number of individuals affected by ulcerative colitis (UC), a chronic inflammatory bowel disease characterized by diffuse, nonspecific inflammation of the colonic mucosa, has been increasing worldwide.^1^ In patients with UC, long-standing disease is a well-established risk factor for the development of UC-associated colorectal neoplasia (UCAN).^2^^,^^3^ Recent improvements in various medical therapies have prolonged disease duration in patients with UC, resulting in a growing population at long-term risk for UCAN. Although population-based studies suggest a decline in the hazard ratio (HR) for UC-associated colorectal cancer diagnosis and cancer related mortality,^4^ UCAN remains a clinically relevant complication, as reported in Japan^5^ and other countries.^6^ Previous multicenter studies conducted in Japan demonstrated a significantly worse prognosis of advanced UCAN vs sporadic colorectal cancer (CRC),^7^ a finding that was confirmed in a recent nationwide study by the Japanese Society for Cancer of the Colon and Rectum (JSCCR).^8^

Despite accumulating data, the clinicopathological features and oncological outcomes of early-onset UCAN, particularly in patients aged <40 years at the time of diagnosis, remain poorly understood. In the context of sporadic CRC, although traditionally considered a disease of older adults, approximately 5%-10% of all cases occur in individuals aged <40 years.^9^ Recent studies reported a global rise in the incidence of early-onset CRC, particularly among individuals aged <50 years.^10^^,^^11^ This trend has been observed in both Western and Asian countries, in which a marked increase in rectal cancers among young men has been reported.^12^ CRC diagnosed in patients aged <40 years, often referred to as “adolescent and young adult CRC,” is typically identified at a more advanced stage and is characterized by more aggressive histological features.^13^ The incidence of such early-onset CRC has also been reported to be increasing in Japan.^14^ Although the epidemiology and tumor biology of early-onset CRC are increasingly being elucidated, comparable data on early-onset UCAN remain scarce. Therefore, clarifying the clinicopathological features and prognostic outcomes of UCAN in younger patients may help improve surveillance strategies and treatment planning, ultimately enhancing the outcomes in this vulnerable subgroup.

This study was a secondary analysis of a nationwide project conducted by the JSCCR, “Database creation and clinicopathological study of gastrointestinal cancer with inflammatory bowel disease.” The JSCCR project represents the largest multicenter cohort of IBD-associated gastrointestinal cancers in Japan. In this secondary analysis, we specifically focused on early-onset UCAN, which, to the best of our knowledge, constitutes the first and largest multicenter study addressing this population in Japan. We investigated the clinicopathological features and oncological outcomes, including cancer-specific survival (CSS) and survival after recurrence (SAR), in patients diagnosed with UCAN before the age of 40 years.

## Materials and methods

### Data collection

We conducted a multicenter retrospective cohort study of data from 1212 patients diagnosed with UCAN during 1983-2020 at 43 institutions that participated in the JSCCR study group. UCAN was defined as the presence of colorectal neoplastic lesions in patients with UC diagnosed pathologically at each participating institution. These lesions were considered to arise in association with chronic intestinal inflammation and were, in principle, located within previously inflamed colonic segments. Premalignant lesions, including low-grade dysplasia (LGD) and high-grade dysplasia (HGD), were included in the definition of UCAN. Based on their age at cancer diagnosis, patients were stratified into the young group (Y group; aged <40 years) and the nonyoung group (NY group; aged ≥40 years). This cutoff was chosen in accordance with previous epidemiological studies defining young-onset CRC as diagnosis in patients aged <40 years and with the definition of adolescent and young adult CRC, which is typically defined as occurring in patients aged <40 years. We also compared the distribution of patients across 2 diagnostic periods (before 2011 and from 2011 onward), because changes in medical practice and surveillance strategies over time may have influenced patient characteristics and outcomes.

We collected and compared data on baseline demographics, age at UC diagnosis, age at UCAN diagnosis, disease duration before carcinogenesis, disease extent defined as the maximum extent of colonic involvement ever recorded during the disease course (pancolitis, left-sided colitis, or proctitis), family history of malignancy, presence of primary sclerosing cholangitis (PSC), and preoperative medical therapies including 5-aminosalicylic acid, corticosteroids, immunomodulators, calcineurin inhibitors (CNIs), biologics within 1 year before surgery, and cytapheresis (CAP) at any point before surgery. Cancer detection mode was classified as surveillance or symptom detection. “Surveillance-detected” UCAN was defined as neoplasia identified during scheduled surveillance colonoscopy performed in asymptomatic patients, regardless of the surveillance interval, which varied among institutions. In contrast, “symptom-detected” UCAN was defined as neoplasia detected following the onset of symptoms that prompted diagnostic colonoscopy. To evaluate baseline mucosal inflammation, we compared the Mayo Endoscopic Score (MES) of both groups (0-1 vs 2-3) assessed at the last colonoscopy before surgery. Surgical procedures were categorized by type (eg, total proctocolectomy with ileal pouch–anal anastomosis [IPAA], including endoscopic treatments and cases of unknown procedures) and approach (minimally invasive surgery [MIS], including laparoscopic and robotic approaches vs open surgery). Histopathological assessments included tumor location (right vs left), pT stage (the pathological T stage defined according to the tumor classification of the TNM staging system, based on the depth of tumor invasion), nodal status, histological subtype (well/moderately differentiated adenocarcinoma [Wel/Mod] vs poorly differentiated adenocarcinoma [Por], mucinous carcinoma [Muc], or signet ring cell carcinoma [Sig]) (when multiple histological subtypes were identified, cases were classified according to the most major histological subtype), and final pathological stage.

The primary outcomes were overall survival, CSS, relapse-free survival (RFS, excluding stage IV cases), and SAR. Hazard ratios (HRs) for CSS were estimated using uni- and multivariate Cox proportional hazard models to identify independent prognostic factors. A subgroup analysis within the Y group was conducted to evaluate the impact of MIS vs open surgery on CSS and RFS. Furthermore, to address potential time-related confounding, an additional subgroup analysis restricted to patients diagnosed in or after 2011 was performed.

### Statistical analysis

All statistical analyses were performed using EZR, a graphical user interface for R software (The R Foundation for Statistical Computing, Vienna, Austria).^15^ Two-sided values of *P < .*05 were considered statistically significant. Categorical variables were compared using the chi-squared test or Fisher’s exact test as appropriate. Continuous variables were analyzed using the Student *t*-test or Mann-Whitney U test depending on the data distribution. Kaplan-Meier survival curves were generated and compared using log-rank tests. Patients without events were censored at the date of last follow-up. Some variables contained missing data due to incomplete information in the retrospective multicenter dataset. Variables with missing values were excluded from the multivariate analyses, and the number of available cases was indicated in each table. Multiple imputation was not performed because the proportion of missing values was limited.

## Results

### Patient population and time trends

The data of a total of 1212 patients diagnosed with UCAN were included in the analysis: 252 (20.8%) and 960 (79.2%) in the Y and NY groups, respectively. When stratified by diagnostic period, more than half of the UCAN cases in both age groups were diagnosed in the most recent decade. However, the proportion of patients in the Y group among the total significantly decreased over time (from 26.7% before 2011 to 17.6% during or after 2011; *P < .*001; patients with missing information on the year of diagnosis [*n* = 17] were excluded from temporal analyses; Table 1).

**Table 1:** Overall patients and time trends by study group.^a^

	All patients, *n* (%)	Patient time trends		*P* value
		**Before 2011, *n*** = **416**	**2011 onward, *n*** = **779**	
**Y group**	252 (20.8%)	111 (26.7%)	137 (17.6%)	<.001
**NY group**	960 (79.2%)	305 (73.3%)	642 (82.4%)	

Abbreviations: NY, not young, age at cancer diagnosis ≥40 years; Y, young, age at cancer diagnosis <40 years.

a Total *n* = 1212. Seventeen patients (1.4%) had missing information on year of UCAN diagnosis and were excluded from temporal trend analyses.

### Clinicopathological features

Compared to the NY group, the Y group showed a significantly earlier age at UC diagnosis (median, 20 vs 39 years; *P < .*001), earlier age at UCAN diagnosis (median, 34 vs 57 years; *P < .*001), and shorter disease duration (median, 14.0 vs 17.0 years; *P < .*001). Although pancolitis was more prevalent in the Y group (81.3% vs 74.8%; *P = .*012), no significant difference was noted in endoscopic disease activity (MES 0-1, 66.1%; vs MES 2-3, 70.7%; *P = .*250). The Y group had a significantly lower rate of a family history of malignancy (10.0% vs 15.8%; *P = .*035) and tended to have a higher prevalence of concomitant (4.4% vs 2.0%; *P = .*056). Regarding preoperative treatment, the Y group was more likely to have undergone CNI (4.4% vs 1.2%; *P = .*003) and CAP (15.3% vs 7.4%; *P = .*003). Symptom-detected UCAN was more common in the Y group (32.9% vs 24.2%), whereas surveillance-detected UCAN was more common in the NY group (59.1% vs 68.5%; *P = .*014; Table 2). The median surveillance interval among patients with available surveillance date information showed no significant difference between the Y and NY groups (0.98 [range, 0.04-5.75] vs 0.95 [range, 0.02-9.35] years; *P = .*424), and most patients underwent colonoscopy within 3 years before diagnosis, with similar proportions in the Y and NY groups (70/74 [94.6%] vs 304/318 [95.6%]).

**Table 2:** Patient characteristics by study group.^a^

	**Y group, *n*** = **252**	**NY group, *n*** = **960**	*P* value
**Sex, male**	168/251 (67%)	583/956 (61%)	.092
**Age at UC diagnosis, y**	20 [7-36]	39 [7-83]	<.001
**Age at UCAN diagnosis, y**	34 [19-39]	57 [40-89]	<.001
**Disease duration, y**	14.0 [0-26.9]	17.0 [0-57.0]	<.001
**Disease extent**			
**Pancolitis**	205/252 (81.3%)	718/960 (74.8%)	
** Left-sided colitis**	41/252 (16.3%)	179/960 (18.6%)	.012
** Proctitis**	6/252 (2.4%)	63/960 (6.5%)	
**Family history (malignancy), (+)**	20/201 (10.0%)	128/809 (15.8%)	.035
**Concomitant PSC, (+)**	10/229 (4.4%)	18/890 (2.0%)	.056
**Preoperative therapy**			
** 5-ASA**	152/181 (84.0%)	577/698 (82.7%)	.740
** Steroid**	71/204 (34.8%)	227/755 (30.1%)	.202
** IM**	26/252 (10.3%)	90/960 (9.4%)	.632
** CNI**	11/252 (4.4%)	12/960 (1.2%)	.003
** Biologics**	8/252 (3.2%)	44/960 (4.6%)	.386
** CAP**	27/177 (15.3%)	49/658 (7.4%)	.003
**Timing of diagnosis**			
** Symptom**	83/252 (32.9%)	232/960 (24.2%)	
** Surveillance**	149/252 (59.1%)	658/960 (68.5%)	.014
** Others**	20/252 (7.9%)	70/960 (7.3%)	
**MES**			
** 0/1**	127/192 (66.1%)	507/717 (70.7%)	.250
** 2/3**	65/192 (33.9%)	210/717 (29.3%)	

Abbreviations: 5-ASA, 5-aminosalicylic acid; CAP, cytapheresis; CNI, calcineurin inhibitor; IM, immunomodulator; MES, Mayo Endoscopic Score; PSC, primary sclerosing cholangitis; UC, ulcerative colitis; UCAN, ulcerative colitis-associated colorectal neoplasia.

a Values are shown as *n/n* (%) or median [range]; *n* indicates the number of available cases for each variable.

### Surgical and pathological findings

In both groups, the most common surgical procedure was total proctocolectomy with hand-sewn IPAA (58.7% of the Y group vs 44.2% of the NY group). The MIS rate, including laparoscopic and/or robotic approaches, was slightly lower in the Y group (39.2% vs 45.5%; *P = .*091), while the postoperative complication rate was slightly higher (35.2% vs 29.6%; *P = .*102). Tumor location and pathological features were largely similar between groups: left-sided tumors predominated in both (83.2% vs 82.6%; *P = .*850), the proportion of tumors with advanced depth (pT ≥ 2) was comparable (50.2% vs 48.4%; *P = .*649), and the lymph node metastasis rate was similar (19.0% vs 21.5%; *P = .*463). No significant intergroup differences were observed in overall pathological stage. However, in the Y group, a significantly higher frequency of poorly differentiated adenocarcinoma, mucinous adenocarcinoma, and signet ring cell carcinoma (Por/Muc/Sig) histology was noted (20.2% vs 13.3%; *P < .*001), while adjuvant chemotherapy was administered more frequently (23.1% vs 18.0%; *P = .*081). The median follow-up duration was significantly longer in the Y group than in the NY group (4.99 [range, 0.04-31.9] vs 4.06 [range, 0-44.0] years; *P = .*011; Table 3).

**Table 3:** Surgical and pathophysiological results by study group.

	**Y group, *n*** = **252**	**NY group, *n*** = **960**	*P* value
**Surgery**			
** Hand-sewn IPAA**	148/252 (58.7%)	424/960 (44.2%)	NA
** Stapled IPAA**	32/252 (12.7%)	115/960 (12.0%)	
** IRA**	6/252 (2.4%)	27/960 (2.8%)	
** TPC**	23/252 (9.1%)	165/960 (17.2%)	
** STC**	15/252 (6.0%)	33/960 (3.4%)	
** Partial resection**	5/252 (2.0%)	86/960 (9.0%)	
** Others^a^**	23/252 (9.1%)	110/960 (11.5%)	
**MIS (+)**	94/240 (39.2%)	392/861 (45.5%)	.091
**Complication (+)**	86/244 (35.2%)	280/945 (29.6%)	.102
**Tumor location**			
** Right side**	41/244 (16.8%)	164/940 (17.4%)	.850
** Left side**	203/244 (83.2%)	776/940 (82.6%)	
**pT stage**			
** cis/1**	108/217 (49.8%)	443/858 (51.6%)	.649
** 2/3/4**	109/217 (50.2%)	415/858 (48.4%)	
**LN metastasis (+)**	43/226 (19.0%)	185/859 (21.5%)	.463
**p Stage**			
** 0**	65/222 (29.3%)	281/869 (32.3%)	.104
** I**	59/222 (26.6%)	255/869 (29.3%)	
** II**	50/222 (22.5%)	141/869 (16.2%)	
** III**	34/222 (15.3%)	157/869 (18.1%)	
** IV**	14/222 (6.3%)	35/869 (4.0%)	
**Histology**			
** LGD/HGD**	26/252 (10.3%)	100/960 (10.4%)	<.001
** Wel/Mod**	150/252 (59.5%)	681/960 (70.9%)	
** Por/Muc/Sig**	51/252 (20.2%)	128/960 (13.3%)	
** Others**	25/252 (9.9%)	51/960 (5.3%)	
**Adjuvant chemotherapy (+)**	56/243 (23.1%)	169/938 (18.0%)	.081
**Follow-up, y**	4.99 [0.04-31.9]	4.06 [0-44.0]	.011

Abbreviations: HGD, high-grade dysplasia; IPAA, ileal-pouch anal anastomosis; IRA, ileorectal anastomosis; cis, carcinoma in situ; LGD, low-grade dysplasia; MIS, minimally invasive surgery; Mod, moderately differentiated adenocarcinoma; Muc, mucinous carcinoma; NA, not applicable; Por, poorly differentiated adenocarcinoma; Sig, signet ring cell carcinoma; STC, subtotal colectomy; TPC, total proctocolectomy; Wel, well-differentiated adenocarcinoma.

Values are shown as *n*/*n* (%) or median [range], *n* indicates the number of available cases for each variable.

a Others include endoscopic resection (endoscopic mucosal resection or endoscopic submucosal dissection).

### Prognostic analysis

**Figure 1:**
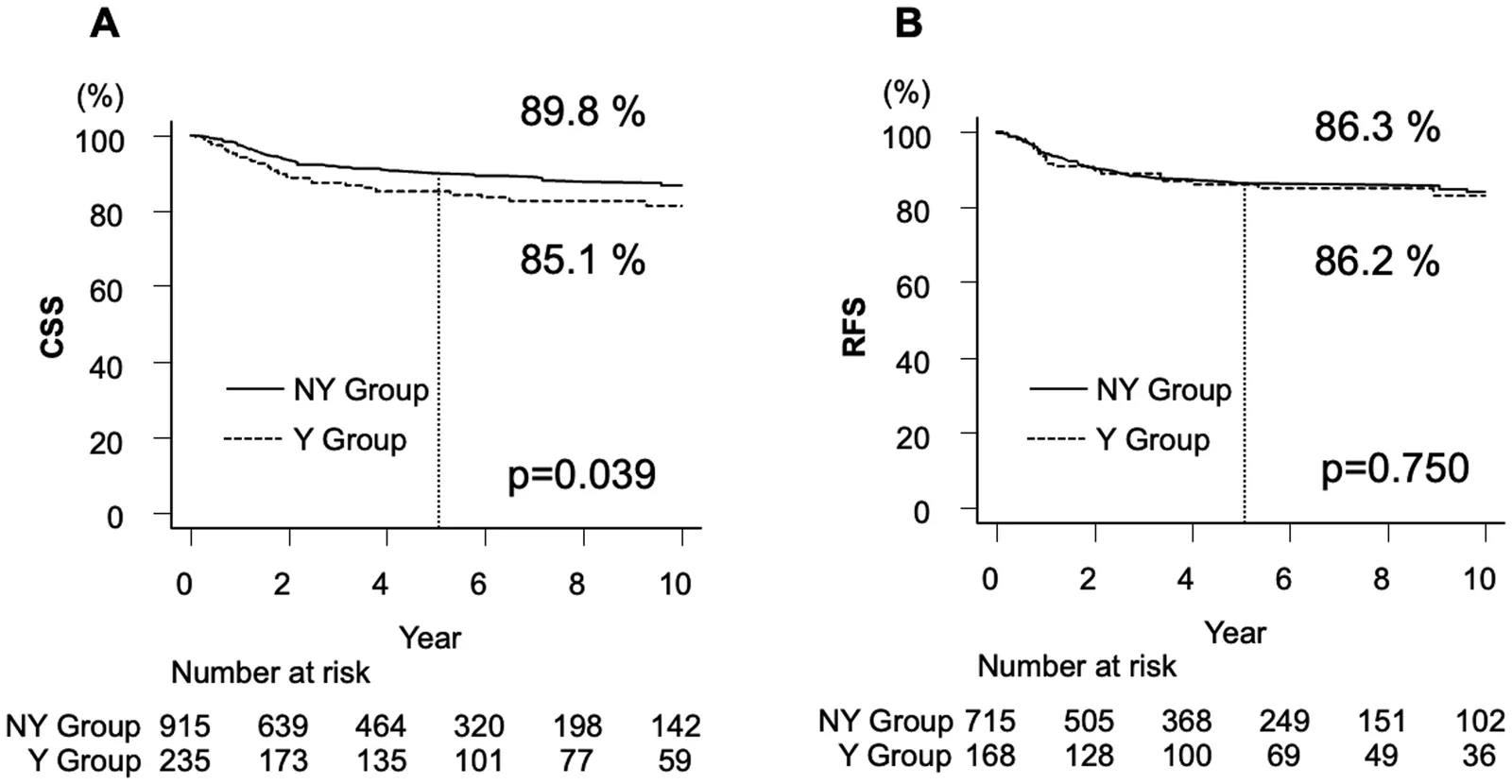
CSS and RFS by age group. Kaplan-Meier curves of (A) cancer-specific survival (CSS) and (B) relapse-free survival (RFS) comparing the Y group (aged <40 years, dashed line) and NY group aged (≥40 years, solid line). NY, not young; Y, young.

**Figure 2:**
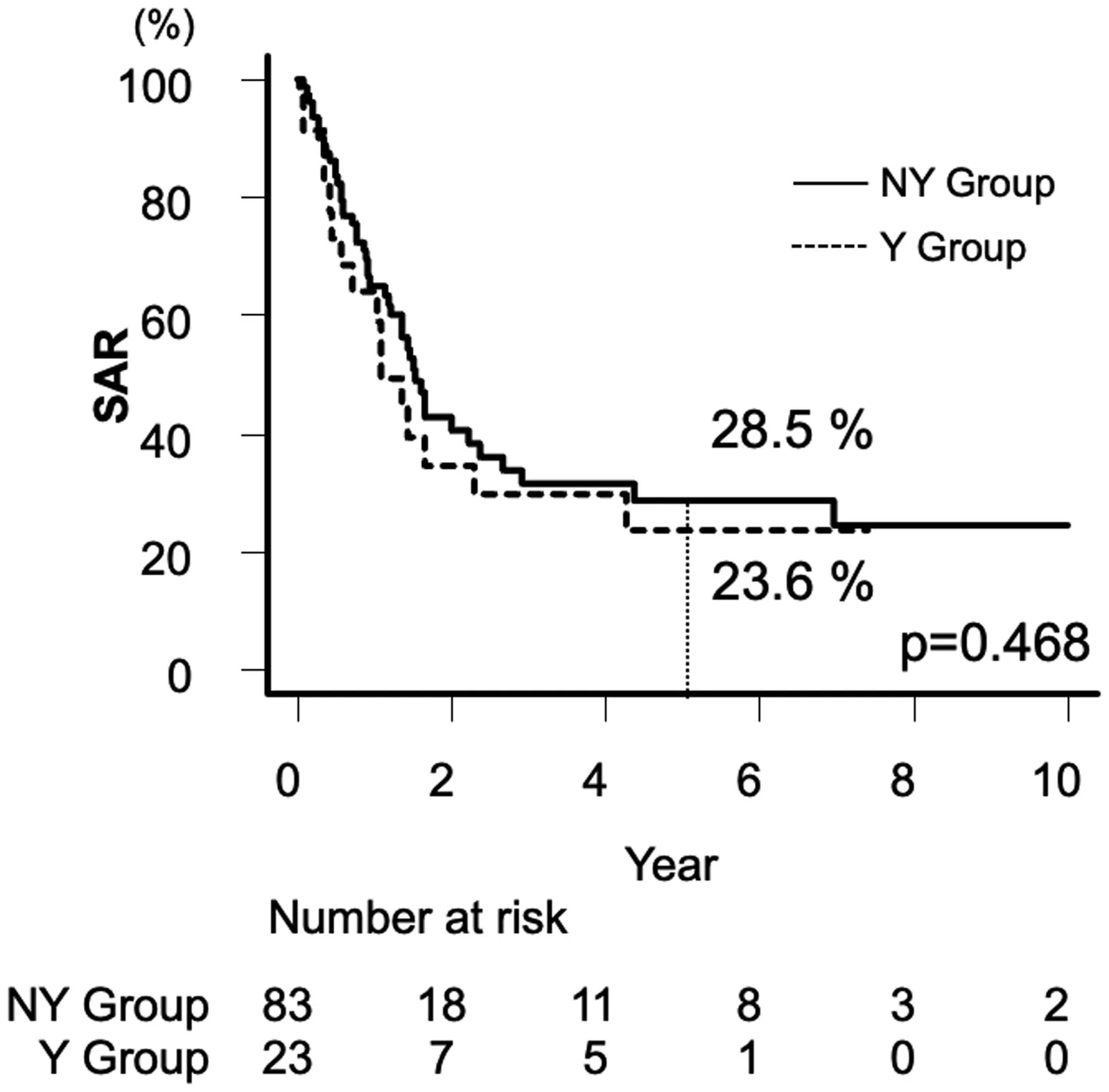
SAR by age group. Kaplan-Meier curve showing survival after recurrence (SAR) of the Y group (aged <40 years, dashed line) vs NY group (aged ≥40 years, solid line). NY, not young; Y, young.

Although OS was associated with a poorer prognosis in the Y group (82.4% vs 87.5% at 5 years; *P = .*087), CSS was significantly lower (85.1% vs 89.8% at 5 years; *P = .*039). RFS was similar between groups (86.2% vs 86.3% for Y group vs NY group, respectively; *P = .*750; Figure 1). The SAR showed poor prognosis in both groups and was comparable (23.6% vs 28.5%; *P = .*468; Figure 2). When stratified by pathological stage, CSS was significantly lower in the Y group for stage II disease (85.3% vs 93.6%, *P = .*030), whereas no statistically significant difference was observed for other stages (Figure S1).

In the univariate analysis, age < 40 years was associated with worse CSS (HR, 1.519; 95% CI, 1.017-2.269; *P = .*041), although result this was not statistically significant on the multivariate analysis (HR, 1.255; 95% CI, 0.784-2.007; *P = .*344). Other risk factors associated with the worse CSS identified in the univariate analysis included left-sided tumor location, advanced pT stage, lymph node metastasis, Por/Muc/Sig histology, and adjuvant chemotherapy usage. Although MIS was associated with a good prognosis, the time trend might have influenced the results because more recent cases were selected for MIS. On the univariate analysis, patients treated after 2011 showed a better prognosis than those treated before 2010 (HR, 0.668; 95% CI, 0.458-0.974; *P = .*036). Therefore, age, tumor location, pT stage, lymph node metastasis, pathology, adjuvant chemotherapy, MIS, and time trends were subjected to multivariate analysis.

The multivariate analysis revealed that the independent predictors of poor CSS were left-sided tumor location (HR, 2.546; 95% CI, 1.166-5.559; *P = .*019), advanced pT stage (HR, 4.519; 95% CI, 1.998-10.23; *P < .*001), lymph node metastasis (HR, 4.833; 95% CI, 2.802-8.337; *P < .*001), and Por/Muc/Sig histology (HR, 2.224; 95% CI, 1.414-3.496; *P < .*001). In contrast, MIS was independently associated with improved CSS (HR, 0.593; 95% CI, 0.356-0.987; *P = .*045). Adjuvant chemotherapy was not an independent prognostic factor (HR, 0.862; 95% CI, 0.536-1.386; *P = .*539), likely reflecting its association with more advanced disease (Table 4).

**Table 4:** Uni- and multivariate analyses of cancer-specific survival.

Patient characteristic	Finding	Univariate analysis			Multivariate analysis		
		HR	95% CI	*P* value	HR	95% CI	*P* value
**Age, y**	<40/≥40	1.519	1.017-2.269	.041	1.255	0.784-2.007	.344
**Sex**	Male/female	1.292	0.879-1.902	.193			
**Disease extent**	Pancolitis/left side, proctitis	0.851	0.562-1.291	.449			
**PSC**	+/−	1.484	0.547-4.030	.439			
**MES**	2, 3/0, 1	1.268	0.787-2.044	.329			
**CAP**	+/−	0.936	0.431-2.034	.867			
**CNI**	+/−	0.394	0.055-2.824	.354			
**Location**	Left/right	1.808	0.970-3.367	.062	2.546	1.166-5.559	.019
**MIS**	+/−	0.368	0.231-0.587	<.001	0.593	0.356-0.987	.045
**pT**	2, 3, 4/is, 1	13.49	6.817-26.7	<.001	4.519	1.998-10.23	<.001
**pN**	+/−	11.52	7.565-17.55	<.001	4.833	2.802-8.337	<.001
**Pathology**	Por, Muc, Sig/Wel, Mod	6.566	4.555-9.465	<.001	2.224	1.414-3.496	<.001
**Adjuvant chemotherapy**	+/−	4.955	3.365-7.298	<.001	0.862	0.536-1.386	.539
**Time trend**	2011 onward/before 2011	0.668	0.458-0.974	.036	0.916	0.591-1.419	.693

Abbreviations: CAP, cytapheresis; CNI, calcineurin inhibitor; is, carcinoma in situ; CSS, cancer-specific survival; MES, Mayo Endoscopic Score; MIS, minimally invasive surgery; Mod, moderately differentiated adenocarcinoma; Muc, mucinous carcinoma; Por, poorly differentiated adenocarcinoma; PSC, primary sclerosing cholangitis; Sig, signet ring cell carcinoma; Wel, well-differentiated adenocarcinoma.

### Subgroup analysis of young patients

**Figure 3:**
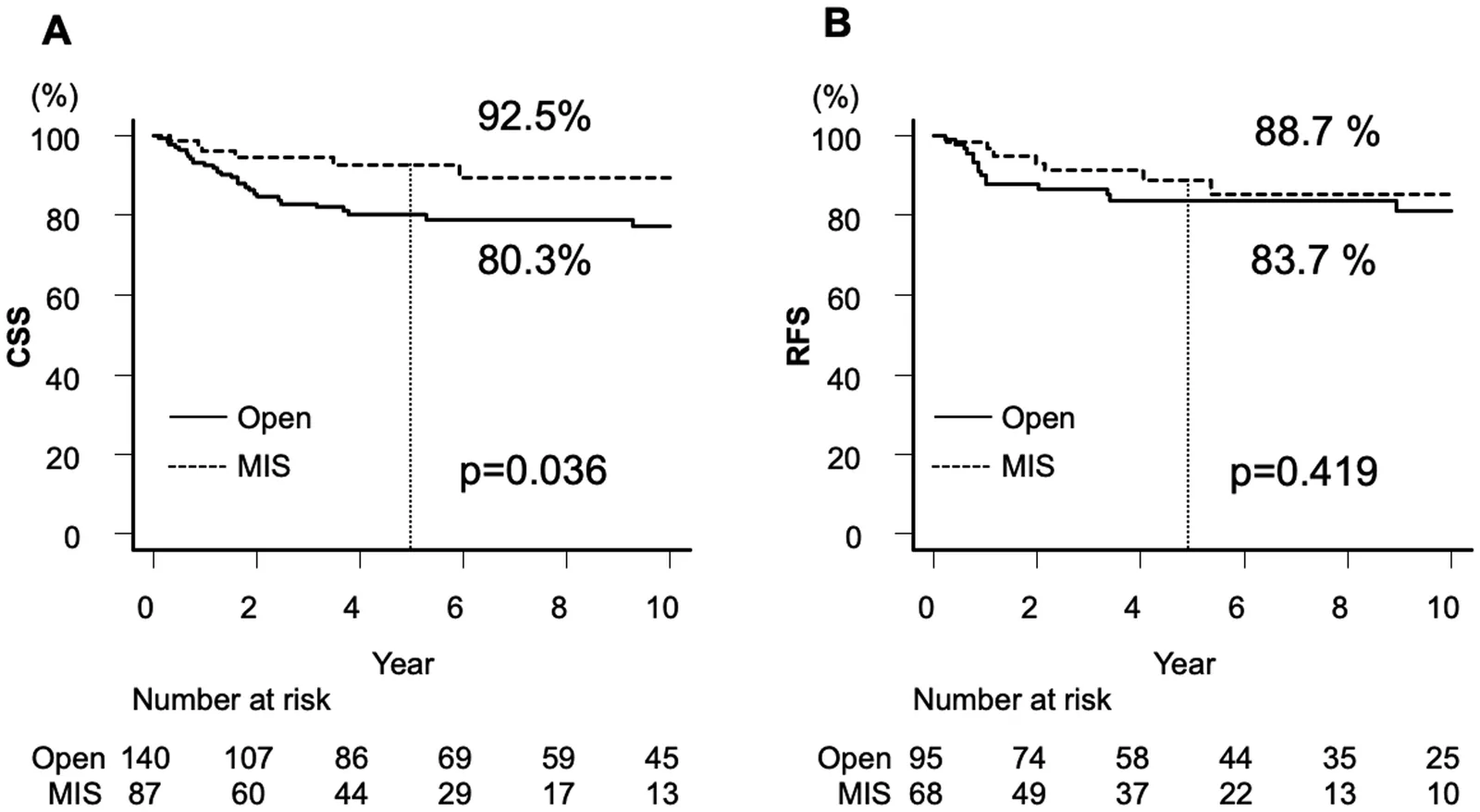
Cancer-specific survival (CSS) and relapse-free survival (RFS) by surgical approach in the Y group (aged <40 years). Y, young. Kaplan-Meier curves of (A) cancer-specific survival (CSS) and (B) relapse-free survival (RFS) by open surgery (solid line) vs minimally invasive surgery (MIS; dashed line) in patients aged <40 years. Twelve patients (4.8%) with missing information on the surgical approach were excluded from the analyses.

Among the patients in the Y group (*n* = 252), those who underwent MIS (*n* = 94) showed significantly better 5-year CSS than those who underwent open surgery (*n* = 146) (92.5% vs 80.3%; *P = .*036), although RFS did not differ significantly (88.7% vs 83.7%, *P = .*419). Patients with missing information on the surgical approach (*n* = 12) were excluded from the analyses Figure 3). Patients in the MIS group had a higher proportion of surveillance-detected tumors (74.5% vs 47.9%, *P < .*001) and were significantly more likely to have undergone treatment in the most recent decade (71.3% vs 43.8%, *P < .*001; Table S1). No significant differences were noted in tumor stage or nodal metastasis between the MIS and open surgery groups. However, patients treated with open surgery were more likely to have advanced-stage disease (Table S2). When stratified by the diagnostic period, although the CSS appeared to improve over time; however, the differences were not statistically significant (5-year: 79.6% before 2011 vs 84.7% in or after 2011-2020; *P = .*325).

### Subgroup analysis of recent 10 years

In the subgroup analysis restricted to patients diagnosed during or after 2011 (*n* = 779), clinicopathological characteristics were largely comparable between the Y and NY groups, including the proportion of surveillance-detected cases (67.9% vs 72.9%, *P = .*475) (Table S3). However, Por/Muc/Sig histology remained significantly more frequent in the Y group (19.7% vs 11.7%, *P = .*004) (Table S4). Although CSS tended to be lower in the Y group, the difference did not reach statistical significance (5-year CSS, 88.7% vs 91.2%; *P = .*104) (Figure S2).

## Discussion

In this large multicenter cohort study of the data for more than 1200 patients with UCAN in Japan, we comprehensively compared the clinicopathological features and oncological outcomes of those diagnosed before vs after 40 years of age. To the best of our knowledge, this is the first and among the largest studies to specifically focus on early-onset UCAN. No previous studies, to our knowledge, evaluated long-term prognostic outcomes over such an extended follow-up period. Our findings highlight the distinct features of young patients with UCAN, particularly those with tumors with aggressive histological features and poor CSS, which may influence clinical management and prognosis.

Young patients account for over 20% of all UCAN cases, suggesting that early-onset UCAN is not uncommon in Japan compared to early-onset sporadic CRC, which comprises 5%-10% of all CRC cases.^9^ Although the absolute number of early-onset UCAN cases increased over time, particularly in the most recent decade, the proportion of young patients slightly decreased in contrast to the global trend of increasing early-onset sporadic CRC.^11^ Notably, early-onset UCAN demonstrated significantly more aggressive histopathological features, such as Por/Muc/Sig, consistent with findings of sporadic early-onset CRC.^10^ Other distinct clinicopathological features included a higher prevalence of pancolitis and shorter interval from UC onset to neoplasia development. These findings may reflect a higher inflammatory burden and a more refractory disease phenotype rather than a direct carcinogenic effect of specific treatments such as CNI or CAP, consistent with previous studies identifying pancolitis as a risk factor for UCAN.^16^ Moreover, the proportion of surveillance-detected cancers was significantly lower in the Y group, with a higher proportion being diagnosed symptomatically. This finding suggests that both delayed detection and aggressive tumor biology may underlie the poor CSS observed in younger patients. Indeed, a previous Japanese multicenter study demonstrated that patients with surveillance lesions had significantly better prognoses than those diagnosed symptomatically.^17^ Therefore, surveillance colonoscopy may help improve the prognosis of patients with early-onset UCAN. Despite the higher rates of poor differentiation and aggressive histology in the Y group, RFS was similar between the age groups, suggesting that adjuvant chemotherapy may have conferred some benefits. However, the SAR was poor in both groups, highlighting the need to prevent recurrence in younger and older patients to improve overall outcomes.

CSS was significantly worse in the Y group, especially among patients with advanced-stage disease. In the multivariate analysis, age itself was not an independent prognostic factor, whereas left-sided tumor location, higher pT stage, nodal metastasis, and poor histological differentiation were significantly associated with a worse prognosis. Although the initiation of adjuvant chemotherapy was associated with a worse CSS on the univariate analysis, this association was not significant after the adjustment for confounding factors, suggesting that chemotherapy served as a surrogate marker for advanced disease rather than determinant of poor prognosis. Interestingly, MIS was associated with better survival even after the adjustment for tumor stage and other factors. Young patients who underwent MIS also demonstrated more favorable CSS than those who underwent open surgery despite comparable tumor stage and nodal status. However, these findings should be interpreted with caution. While MIS is generally expected to improve short-term outcomes, such as reduced blood loss and fewer postoperative complications,^18^ its potential influence on long-term oncological outcomes remains uncertain and controversial. Notably, MIS was more frequently performed in recent years, when surveillance strategies were more widely adopted, resulting in a higher proportion of earlier-stage disease, including LGD or HGD, in the MIS group. This time-related trend may partly explain the more favorable survival outcomes observed after MIS rather than a direct causal effect of the surgical approach itself. This trend likely reflects recent changes in clinical practice, including the broader adoption of MIS for UC.^19^ These temporal and clinical factors may have contributed to the observed association. Taken together, although our findings suggest the possible benefits of the MIS, the association between MIS and improved CSS in the present study should be interpreted with caution and should not be considered evidence of a causal relationship. Rather, this finding likely reflects selection bias, temporal changes in surveillance and surgical practice, and differences in disease stage at diagnosis, including the inclusion of dysplasia. Further large-scale prospective studies are needed to clarify the impact of MIS on long-term outcomes in this population.

Interestingly, despite the earlier-onset UC and shorter disease duration before neoplasia development in group Y, these patients had a lower prevalence of a family history of malignancy. This finding suggests that early-onset UCAN may not necessarily reflect inherited cancer syndromes but rather distinct pathogenic pathways involving inflammation-induced carcinogenesis in the younger colonic mucosa. It has been generally reported that *TP53* mutations occur in the early phase of inflammation-induced carcinogenesis, whereas *KRAS* mutations are more commonly observed in the early stages of the sporadic CRC pathway.^20–22^ However, molecular data such as *TP53* or *KRAS* mutation status were unavailable in this study because of its retrospective design. Future investigations incorporating such molecular profiles will be important to advance our understanding of the tumorigenic mechanisms underlying early-onset UCAN. Although the prevalence of concomitant PSC tended to be higher in group Y, this difference was not statistically significant. Given that PSC is a known risk factor of UCAN,^23^ careful attention should be paid to this comorbidity, particularly in younger patients with UCAN.

This study has several limitations. First, it was a retrospective study; thus, selection bias and potential confounders are unavoidable. Second, although we utilized data from multiple high-volume centers across Japan, most participating institutions were surgical centers; therefore, patients with nonoperative cases, including those managed exclusively with endoscopic treatment, may be underrepresented in this cohort. Furthermore, institutional variability in surveillance protocols and surgical indications may have influenced the results. In addition, the follow-up period varied among institutions, and the median follow-up duration was less than 5 years, which may limit the interpretation of long-term survival outcomes. Third, due to the retrospective nature of the study, molecular and genetic data were unavailable, limiting our ability to explore the biological underpinnings of early-onset UCAN. Finally, although we adjusted for major clinicopathological factors in the multivariate analyses, residual confounding factors could not be entirely excluded. Despite these limitations, this study offers novel insights into the clinical features and prognosis of early-onset UCAN, a rare condition that may be difficult to prospectively investigate.

In summary, early-onset UCAN exhibits distinct clinicopathological and prognostic features compared with that in older patients. These findings underscore the need for age-tailored strategies for surveillance, surgical planning, and perioperative management to improve outcomes in this vulnerable subgroup.

## Conclusion

Approximately one-fifth of all UCAN cases occur in patients aged <40 years and are characterized by an earlier onset, more extensive colonic inflammation, and histologically aggressive features. Although RFS was comparable between age groups, CSS was significantly worse in early-onset cases. The potential role of MIS and enhanced surveillance strategies in improving the prognosis of younger patients warrants further prospective investigations. Improving early detection and optimizing surgical management may be crucial to enhancing the outcomes of this increasingly important patient population.

## Supplementary Material

izag069_Supplementary_Data

## Data Availability

The data underlying this article will be shared after all the analyses are completed on reasonable request to the corresponding author.
