# Supplementary material for: Clinicopathological features and cancer-specific survival in young patients with ulcerative colitis–associated colorectal neoplasia: a nationwide cohort study in Japan
Source: Inflamm Bowel Dis. 2026 May 19;32(9):1750–9. doi: 10.1093/ibd/izag069 (PMC13533184; doi:10.1093/ibd/izag069)
Supplement: Supplementary file 1 [file izag069_supplementary_data.docx]

|  |  | **Open** | **MIS** | **P value** |
| --- | --- | --- | --- | --- |
|  |  | **N = 146** | **N = 94** |  |
| **SEX** | **Male** | 96/146 (65.8%) | 64/93 (68.8%) | 0.673 |
| **Age at UC diagnosis** |  | 19 [8-36] | 20 [7-34] | 0.393 |
| **Age at UCAN diagnosis** |  | 34 [20-39] | 34.5 [19-39] | 0.722 |
| **Disease duration (years)** |  | 14.4 [0-26.9] | 13.0 [0.75-25.0] | 0.086 |
| **Disease extent** | **Pancolitis** | 124/146 (84.9%) | 75/94 (79.8%) |  |
|  | **Left-sided colitis** | 19/146 (13.0%) | 17/94 (18.1%) | 0.507 |
|  | **Proctitis** | 3/146 (2.1%) | 2/94 (2.1%) |  |
| **Family history (malignancy)** | **(+)** | 10/108 (9.3%) | 9/82 (11.0%) | 0.808 |
| **Concomitant PSC** | **(+)** | 3/125 (2.4%) | 5/92 (5.4%) | 0.288 |
| **Preoperative therapy** | **5-ASA** | 70/91 (76.9%) | 73/80 (91.2%) | 0.013 |
|  | **Steroid** | 43/114 (37.7%) | 26/80 (32.5%) | 0.543 |
|  | **IM** | 13/146 (8.9%) | 12/94 (12.8%) | 0.389 |
|  | **CNI** | 3/146 (2.1%) | 7/94 (7.4%) | 0.051 |
|  | **Biologics** | 4/146 (2.7%) | 3/94 (3.2%) | 1 |
|  | **CAP** | 15/100 (15.0%) | 11/69 (15.9%) | 1 |
| **Timing of diagnosis** | **Symptom** | 60/146 (41.1%) | 20/94 (21.3%) | <0.001 |
|  | **Surveillance** | 70/146 (47.9%) | 70/94 (74.5%) |  |
|  | **Others** | 16/146 (11.0%) | 4/94 (4.3%) |  |
| **MES*** | **0 / 1** | 72/110 (65.5%) | 47/73 (64.4%) | 1 |
|  | **2 / 3** | 38/110 (34.5%) | 26/73 (35.6%) |  |
| **Timing of diagnosis, year** | **Before 2011** | 82/146 (56.2%) | 27/94 (28.7%) | <0.001 |
|  | **2011 onward** | 64/146 (43.8%) | 67/94 (71.3%) |  |

**Supplementary Tables**

**Supplementary Table 1. Characteristics of patients treated with open versus MIS in Y group**

5-ASA, 5-aminosalicylic acid; CAP, cytapheresis; CNI, calcineurin inhibitor; IM, immunomodulator; MES, Mayo Endoscopic Score; PSC, primary sclerosing cholangitis; UC, ulcerative colitis; UCAN, ulcerative colitis-associated colorectal neoplasia

Values are shown as n/N (%) or median [range]. N indicates the number of available cases for each variable.

|  |  | **Open** | **MIS** | **P value** |
| --- | --- | --- | --- | --- |
|  |  | **N = 146** | **N = 94** |  |
| **Surgery** | **Hand-sewn IPAA** | 92/146 (63.0%) | 54/94 (57.4%) | NA |
|  | **Stapled IPAA** | 6/146 (4.1%) | 26/94 (27.7%) |  |
|  | **IRA** | 3/146 (2.1%) | 3/94 (3.2%) |  |
|  | **TPC** | 21/146 (14.4%) | 2/94 (2.1%) |  |
|  | **STC** | 11/146 (7.5%) | 4/94 (4.3%) |  |
|  | **Partial resection** | 2/146 (1.4%) | 3/94 (3.2%) |  |
|  | **Others** | 11/146 (7.5%) | 2/94 (2.1%) |  |
| **Complication** | **(+)** | 45/143 (31.5%) | 39/92 (42.4%) | 0.096 |
| **Tumor Location** | **Right side** | 22/144 (15.3%) | 18/91 (19.8%) | 0.379 |
|  | **Left side** | 122/144 (84.7%) | 73/91 (80.2%) |  |
| **pT stage** | **is / 1** | 63/133 (47.4%) | 40/77 (51.9%) | 0.568 |
|  | **2 / 3 / 4** | 70/133 (52.6%) | 37/77 (48.1%) |  |
| **LN metastasis** | **(+)** | 30/133 (22.6%) | 12/87 (13.8%) | 0.117 |
| **pStage** | **0** | 38/134 (28.4%) | 24/80 (30.0%) | 0.287 |
|  | **I** | 30/134 (22.4%) | 27/80 (33.8%) |  |
|  | **II** | 32/134 (23.9%) | 17/80 (21.2%) |  |
|  | **III** | 24/134 (17.9%) | 9/80 (11.2%) |  |
|  | **IV** | 10/134 (7.5%) | 3/80 (3.8%) |  |
| **Histology** | **LGD / HGD** | 8/146 (5.5%) | 15/94 (16.0%) | 0.071 |
|  | **Wel / Mod** | 92/146 (63.0%) | 53/94 (56.4%) |  |
|  | **Por / Muc / Sig** | 32/146 (21.9%) | 18/94 (19.1%) |  |
|  | **Others** | 14/146 (9.6%) | 8/94 (8.5%) |  |

**Supplementary Table 2. Surgical and pathophysiological results of patients in Y group**

HGD, high-grade dysplasia; IPAA, ileal-pouch anal anastomosis; IRA, ileorectal anastomosis; is, carcinoma in situ; LGD, low-grade dysplasia; MIS, minimally invasive surgery; Mod, moderately differentiated adenocarcinoma; Muc, mucinous carcinoma; Por, poorly differentiated adenocarcinoma; Sig, signet ring cell carcinoma; STC, subtotal colectomy; TPC, total proctocolectomy; Wel, well-differentiated adenocarcinoma

Values are shown as n/N (%). N indicates the number of available cases for each variable.

**Supplementary Table 3. Patient characteristics by age group among patients diagnosed in or after 2011**

|  |  | **Y group** | **NY group** | **P value** |
| --- | --- | --- | --- | --- |
|  |  | **N = 137** | **N = 642** |  |
| **Sex** | **Male** | 87/136 (64.0%) | 397/638 (62.2%) | 0.770 |
| **Age at UC diagnosis** |  | 20 [7-36] | 39 [7-83] | <0.001 |
| **Age at UCAN diagnosis** |  | 34 [19-39] | 58 [40-89] | <0.001 |
| **Disease duration (years)** |  | 14.0 [0-26.5] | 17.9 [0-57.0] | <0.001 |
| **Disease extent** | **Pancolitis** | 115/134 (85.8%) | 486/627 (77.5%) |  |
|  | **Left-sided colitis** | 18/134 (13.4%) | 121/627 (19.3%) | 0.155 |
|  | **Proctitis** | 1/134 (0.7%) | 20/627 (3.2%) |  |
| **Family history (malignancy)** | **(+)** | 10/118 (8.5%) | 83/556 (14.9%) | 0.077 |
| **Concomitant PSC** | **(+)** | 5/135 (3.7%) | 13/611 (2.1%) | 0.346 |
| **Preoperative therapy** | **5-ASA** | 94/111 (84.7%) | 428/511 (83.8%) | 0.887 |
|  | **Steroid** | 37/114 (32.5%) | 139/527 (26.4%) | 0.203 |
|  | **IM** | 25/137 (18.2%) | 78/642 (12.1%) | 0.070 |
|  | **CNI** | 8/137 (5.8%) | 7/642 (1.1%) | 0.002 |
|  | **Biologics** | 8/137 (5.8%) | 41/642 (6.4%) | 1 |
|  | **CAP** | 20/101 (19.8%) | 31/461 (6.7%) | <0.001 |
| **Timing of diagnosis** | **Symptom** | 35/137 (25.5%) | 138/642 (21.5%) |  |
|  | **Surveillance** | 93/137 (67.9%) | 468/642 (72.9%) | 0.475 |
|  | **Others** | 9/137 (6.6%) | 36/642 (5.6%) |  |
| **MES** | **0 / 1** | 75/110 (68.2%) | 363/513 (70.8%) | 0.646 |
|  | **2 / 3** | 35/110 (31.8%) | 150/513 (29.2%) |  |

5-ASA, 5-aminosalicylic acid; CAP, cytapheresis; CNI, calcineurin inhibitor; IM, immunomodulator; MES, Mayo Endoscopic Score; PSC, primary sclerosing cholangitis; UC, ulcerative colitis; UCAN, ulcerative colitis-associated colorectal neoplasia

Values are shown as n/N (%) or median [range]. N indicates the number of available cases for each variable.

**Supplementary Table 4. Surgical and pathophysiological results by age group among patients diagnosed in or after 2011**

|  |  | **Y group** | **NY group** | **P value** |
| --- | --- | --- | --- | --- |
|  |  | **N = 137** | **N = 642** |  |
| **Surgery** | **Hand-sewn IPAA** | 83/137 (60.6%) | 283/642 (44.1%) | NA |
|  | **Stapled IPAA** | 18/137 (13.1%) | 78/642 (12.1%) |  |
|  | **IRA** | 3/137 (2.2%) | 13/642 (2.0%) |  |
|  | **TPC** | 10/137 (7.3%) | 113/642 (17.6%) |  |
|  | **STC** | 8/137 (5.8%) | 23/642 (3.6%) |  |
|  | **Partial resection** | 3/137 (2.2%) | 52/642 (8.1%) |  |
|  | **Others*** | 12/137 (8.8%) | 80/642 (12.5%) |  |
| **MIS** | **(+)** | 67/131 (51.1%) | 306/569 (53.8%) | 0.628 |
| **Complication** | **(+)** | 54/136 (39.7%) | 181/635 (28.5%) | 0.014 |
| **Tumor location** | **Right side** | 27/134 (20.1%) | 116/631 (18.4%) | 0.627 |
|  | **Left side** | 107/134 (79.9%) | 515/631 (81.6%) |  |
| **pT stage** | **is / 1** | 59/113 (52.2%) | 295/567 (52.0%) | 1 |
|  | **2 / 3 / 4** | 54/113 (47.8%) | 272/567 (48.0%) |  |
| **LN metastasis** | **(+)** | 24/122 (19.7%) | 113/558 (20.3%) | 0.463 |
| **pStage** | **0** | 36/116 (31.0%) | 190/570 (33.3%) | 0.187 |
|  | **I** | 28/116 (24.1%) | 165/570 (28.9%) |  |
|  | **II** | 26/116 (22.4%) | 100/570 (17.5%) |  |
|  | **III** | 18/116 (15.5%) | 98/570 (17.2%) |  |
|  | **IV** | 8/116 (6.9%) | 17/570 (3.0%) |  |
| **Histology** | **LGD / HGD** | 16/137 (11.7%) | 75/642 (11.7%) | 0.004 |
|  | **Wel / Mod** | 80/137 (58.4%) | 460/642 (71.7%) |  |
|  | **Por / Muc / Sig** | 27/137 (19.7%) | 75/642 (11.7%) |  |
|  | **Others** | 14/137 (10.2%) | 32/642 (5.0%) |  |
| **Adjuvant**  **chemotherapy** | **(+)** | 31/132 (23.5%) | 117/636 (18.4%) | 0.183 |
| **Follow-up (years)** |  | 3.39 [0.04-8.58] | 2.87 [0-8.93] | 0.355 |

HGD, high-grade dysplasia; IPAA, ileal-pouch anal anastomosis; IRA, ileorectal anastomosis; is, carcinoma in situ; LGD, low-grade dysplasia; MIS, minimally invasive surgery; Mod, moderately differentiated adenocarcinoma; Muc, mucinous carcinoma; Por, poorly differentiated adenocarcinoma; Sig, signet ring cell carcinoma; STC, subtotal colectomy; TPC, total proctocolectomy; Wel, well-differentiated adenocarcinoma

*Others include endoscopic resection (endoscopic mucosal resection or endoscopic submucosal dissection).

Values are shown as n/N (%) or median [range]. N indicates the number of available cases for each variable.

**Supplementary Figure Legends**

**Supplementary Figure 1. CSS by age group and pathological stage**

Kaplan–Meier curve of cancer-specific survival (CSS) comparing the Y group (<40 years, dashed line) and NY group (≥40 years, solid line) by each pathological stage (A: Stage 0; B: Stage I; C: Stage II; D: Stage III; E: Stage IV).

**Supplementary Figure 2. CSS by age group among patients diagnosed in or after 2011**

Kaplan–Meier curve of cancer-specific survival (CSS) comparing the Y group (<40 years, dashed line) and NY group (≥40 years, solid line), restricted to patients diagnosed in or after 2011.

**Supplementary Figure 1. CSS by age group and pathological stage**

**Supplementary Figure 2. CSS by age group among patients diagnosed in or after 2011**
